# A Narrative Review of Stroke of Cortical Hand Knob Area

**DOI:** 10.3390/medicina60020318

**Published:** 2024-02-13

**Authors:** Jamir Pitton Rissardo, Vishnu Vardhan Byroju, Sushni Mukkamalla, Ana Letícia Fornari Caprara

**Affiliations:** 1 Neurology Department, Cooper University Hospital, Camden, NJ 08103, USA; jamirrissardo@gmail.com (J.P.R.); byroju-vishnu@cooperhealth.edu (V.V.B.); 2 Cooper Medical School of Rowan University, Camden, NJ 08103, USA; mukkamalla-sushni@cooperhealth.edu; 3 Medicine Department, Federal University of Santa Maria, Santa Maria 97105-900, RS, Brazil

**Keywords:** hand knob, hand paralysis, wrist drop, monoparesis, isolated hand paresis, hand motor cortex infarction, precentral gyrus

## Abstract

The cortical hand knob region of the brain is a knob-like segment of the precentral gyrus, projecting into the middle genu of the central sulcus. This anatomic landmark is responsible for intricate control of hand motor movements and has often been implicated in motor weakness following stroke. In some instances, damage to this area has been mistaken for peripheral causes of hand weakness. Our article aims to consolidate clinically relevant information on the cortical hand knob area in a comprehensive review to guide clinicians regarding diagnosis and treatment strategies. We conducted a systematic search within the Medline/PubMed database for reports of strokes in the cortical hand knob region. All studies were published electronically up until December 2023. The search was conducted using the keyword “hand knob”. A total of 24 reports containing 150 patients were found. The mean and median ages were 65 and 67 years, respectively. Sixty-two percent of the individuals were male. According to the TOAST criteria for the classification of the stroke, 59 individuals had a stroke due to large-artery atherosclerosis, 8 had small-vessel occlusion, 20 had cardioembolism, 25 were determined, and 38 were undetermined. The most common etiologies for stroke in the hand knob area can be attributed to large vessel occlusions, small vessel occlusions, or cardioembolism. Presentations following damage to this area can mimic ulnar, median, or radial neuropathy as well. Our comprehensive review serves as a resource for recognizing and managing stroke in the cortical hand knob area.

## 1. Introduction

Stroke remains the world’s second-largest cause of death and contributes significantly to the global burden of diseases [1]. Approximately 90 percent of strokes have modifiable risk factors [2]. Data has shown that men are more affected than women when analyzing the incidence of ischemic strokes secondary to vascular factors, and women tend to present with atypical symptoms, resulting in delayed care [3].

The early recognition of symptoms suggestive of a cerebrovascular accident is important to initiate treatment and decrease disabilities. Management of stroke includes rehabilitation and treatment of post-stroke complications, which encompasses not only physical disabilities, but also higher levels of anxiety and depression. The time window of stroke presentation is essential and determines management and outcomes [4].

Stroke can present with focal and nonfocal symptoms. Focal deficits are neurological symptoms and signs that can be localized to a defined anatomical location of the brain [5]. Nonfocal symptoms cannot be pinpointed to a specific localization in the brain and can be harder to recognize. Generalized weakness and confusion are the most commonly reported nonfocal symptoms [6].

There should be a high index of suspicion for stroke in patients presenting with the aforementioned signs and symptoms, especially if they have comorbidities or genetic risk factors, such as cerebral arteriopathy, factor V Leiden mutation, and sickle cell anemia [7].

Another approach to classify strokes is based on the circulation area affected. Posterior circulation is involved in one out of five strokes, and diagnosis is often challenging. Patients can present with visual symptoms, loss of balance, confusion, vertigo, dysarthria, and dizziness [8]. Moreover, the National Institutes of Health Stroke Scale is the standardized assessment tool for stroke, but its sensitivity in posterior circulation strokes is low [9].

The most common stroke mimics include peripheral vertigo, accounting for twenty-five percent of stroke mimics, seizures with Todd’s paralysis as presenting signs, syncope, and functional disorders. Metabolic etiologies such as hypoglycemia, hypokalemia, and hyponatremia can present with confusion and lethargy and often trigger stroke code activation [9].

**Figure 1:**
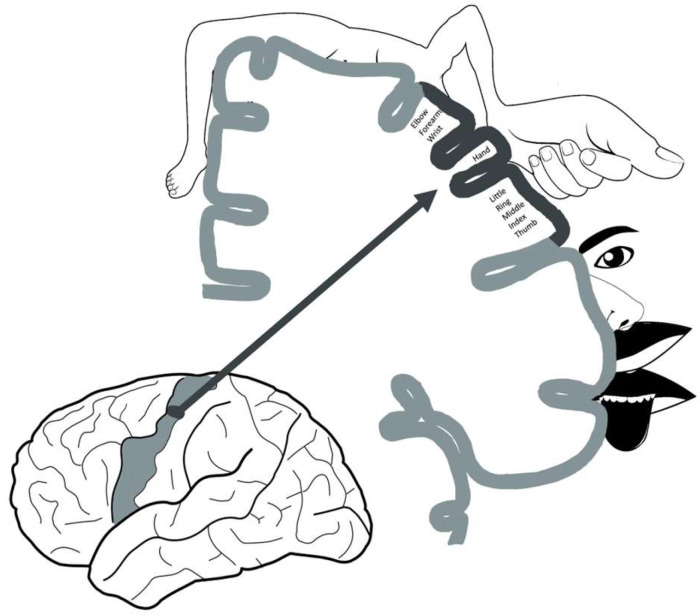
Schematic diagram of the homunculus, in detail, and the hand motor cortex. The hand motor cortex is located in the superior aspect of the precentral gyrus, in the “middle knee” region of the central sulcus.

In this context, while the homunculus theory has existed since the 1930s, it was in 1995 that, for the first time, documentation of a distinct area responsible for hand function was established. An omega/epsilon-shaped region at the junction point between the precentral sulcus and central sulcus on the axial plane was noted to be involved in the motor function of the hand in functional brain MRI. Since then, it also served as a reliable landmark for neuroanatomical localization. Yousry et al. in 1997 reported the first patient that had a circumscribed infarct of the “hand knob area” who presented with isolated contralateral arm weakness without sensation changes [10]. Multiple case reports and series have been published since describing the same observation (Figure 1). It is noteworthy that the most frequent cause of isolated hand weakness is peripheral neuropathy and stroke is only rarely suspected in these patients. Hand knob stroke can present similarly to radial, median, or ulnar nerve injury and without the classic pyramidal or cortical signs. In this way, patients with hand knob stroke are at high risk of being misdiagnosed and receiving inadequate treatment. The lack of investigation of the underlying etiology of the stroke could contribute to improper management of modifiable risk factors such as diabetes, hyperlipidemia, obesity, and hypertension, which could increase the risk of future ischemic events and disabilities. Understanding the hand knob region, its implications, and its role in stroke can further guide rehabilitation efforts and future patient care. Our paper aims to consolidate clinically relevant information on the cortical hand knob area in a comprehensive review to guide clinicians and further treatment strategies.

## 2. Materials and Methods

We searched the Medline/PubMed database to locate existing reports on stroke of the cortical hand knob area published until December 2023 in electronic form. The search term was “hand knob”. The search term was: (“hand” (MeSH Terms) OR “hand” (All Fields)) AND “knob” (All Fields)”, which yielded 256 results.

## 3. Results

### 3.1. Overview

A total of 24 reports containing 150 patients were found (Table 1). The mean and median ages were 65 and 67 years, respectively (range = 30 to 106 years). A total of 62 percent (93 of 150) of the individuals were male. According to the TOAST criteria for the classification of the stroke, 59 individuals had a stroke due to large-artery atherosclerosis, 8 had small-vessel occlusion, 20 had cardioembolism, 25 were determined, and 38 were undetermined.

### 3.2. Epidemiology

Less than one percent of strokes reported occur in the hand knob region, the precentral gyrus of the motor cortex. Some of the risk factors reported include age, sex, and vascular pathologies. Also, the literature points to atheroembolism and cardioembolism as the most common causes, although hemorrhagic strokes in the hand knob areas have been reported. The majority of the cases of hand knob stroke have a good functional outcome secondary to neuronal plasticity, and the risk of recurrence is low. The rarity of the presentation poses challenges in terms of data acquisition regarding the clinical course [17].

The clinical features of hand knob stroke are mild and functional recovery is good. This delays the presentation of the patient, and, added to this, physicians might not be familiar with it and misdiagnose it as a peripheral neuropathy. Imaging findings might not be read accurately as the area of the stroke is small, and the pretest probability of stroke would be low. These factors contribute to the low prevalence of reported hand knob strokes, although the actual number of cases may be higher [23]. Additionally, lack of cortical, pyramidal, and cerebellar symptoms results in evaluating physicians favoring a peripheral source. The mean age of diagnosis of hand knob stroke is 56–71 years old, and data is inconclusive about gender distribution. The term pseudo-peripheral nerve palsy is used to describe this presentation. However, patients often have vascular risk factors favoring the thought process of an alternate diagnosis [16].

Wang et al. reported that hyperhomocysteinemia was the most common risk factor for hand knob infarction [16], while Orosz et al. considered that hypertension was the most common [15]. This last finding was also found by Zhang et al., who observed that hypertension was the most frequent risk factor in patients with hand knob stroke, followed by hyperlipidemia and hyperhomocysteinemia [23]. The table describes the incidence of hand knob stroke in different epidemiological studies (Table 2).

### 3.3. Neuroanatomy and Neuroimaging

The precentral knob is a knob-like segment of the precentral gyrus projecting to the middle genu of the central sulcus that is known to be a reliable anatomic landmark for the motor hand area. Injuries to this area can be caused by various reasons, including stroke, traumatic brain injuries, infections, or tumors. Damage to the precentral knob can result in significant motor impairment to the contralateral hands and fingers. The precentral knob is arranged in a somatotopic fashion where various parts of the hand can be mapped to specific locations, which in turn allows us to localize the area of damage resulting in the motor deficit [26].

**Figure 2:**
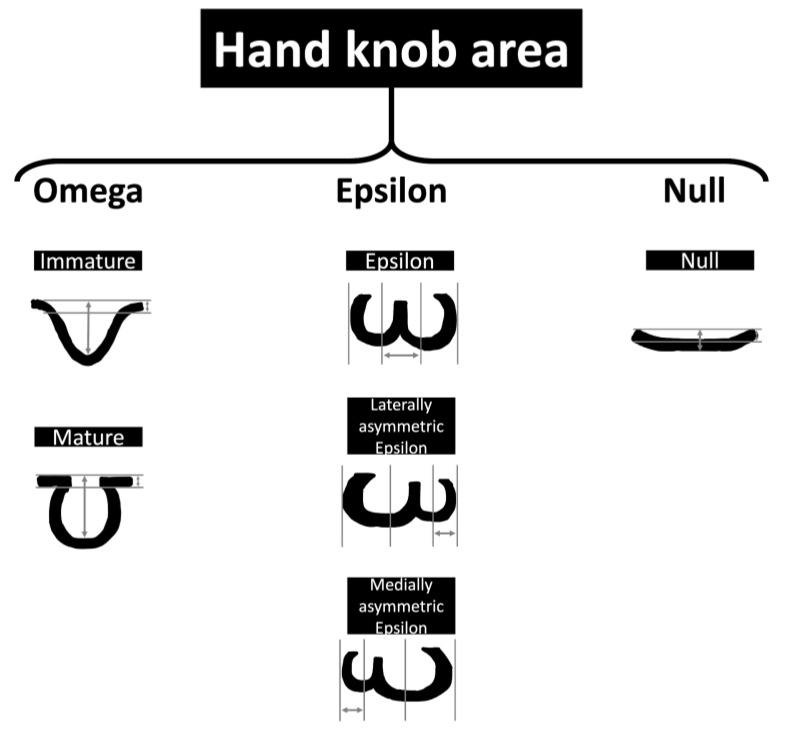
Schematic representation of the hand motor cortex variances. The measurements represent the lengths used in neuroimaging studies to assess the hand knob area.

The hand motor cortex area has been useful in targeting the treatment of multiple neurological conditions such as stroke, amyotrophic lateral sclerosis, brain tumors in adults, and spasticity secondary to cerebral palsy in children (using transcranial magnetic stimulation). The hand knob area has been described consistently as an omega-shaped structure in the axial plane, and this description remains widely accepted [27]. A hook-like structure in the sagittal plane is also accepted. Six variations of anatomical appearance, including epsilon, mediately asymmetric epsilon, laterally asymmetric epsilon, null, mature omega, and immature omega, have been reported in the literature (Figure 2). These variations describe adult brain imaging findings, and when developing brains are considered, immature omega variants are often reported [28].

The hand knob area of the premotor cortex has been studied by Francis et al. using microelectrode recordings, and it was found that face, arm, leg, and head movements are intermixed in the hand knob area. These findings facilitate the decoding of movements using brain-computer interfaces and essentially provide a compositional code of brain motor function. Clinically, this can be helpful for the transfer of motor skills across limbs (for example, analogous movements of hand grasping and toe curling), as was studied in tetraplegic patients. This study represents the ongoing importance of the hand knob area in functional neuroscience [29]. Animal studies have also demonstrated that the caudal region of the hand knob area controls simple movements, and the rostral region is involved in choosing movements through different corticospinal connections and networks, demonstrating the complex architecture of the hand knob area [30].

#### 3.3.1. Neuroanatomy Development

Hand weakness, in the absence of other deficits, can be due to central or peripheral lesions. It is oftentimes misdiagnosed as damage to peripheral nerves, notably the median or ulnar nerve. Motor control and movement planning can be further localized to specific regions within the frontal lobe and are subdivided into M1 (primary motor cortex located in the precentral gyrus of the frontal lobe), M2 (supplementary motor area responsible for coordination and planning of movements, primarily bilateral movements), and M3 (premotor cortex, responsible for preparing the body for movements). Most hand weakness due to central lesions is localized to the M1 knob area. The knob area is particularly associated with movements of the fingers and hand with distinct regions that correspond to individual fingers or specific hand functions. In a study conducted by Chen et al. in 2006, the clinical and radiological profiles of patients were studied to better understand the location of lesions resulting in isolated motor hand weakness in those with cerebral infarction. The results of the study demonstrated that isolated hand weakness in cerebral infarctions can be localized to areas other than the M1 knob area, which has been the area most implicated in these deficits. Other locations emphasized in this study include infarctions of the angular gyri due to stenosis of the internal carotid artery [31].

An interesting observation has been made by Hanakawa et al. in 2005. While contralateral precentral gyrus activity was known to be associated with movements of the arm, the ipsilateral precentral gyrus played a part in complex movements and stroke recovery. Previous reports described activity in the right precentral gyrus during movement of the left arm, whereas the left precentral gyrus activity was observed during ipsilateral and contralateral hand movements. Overall, contralateral precentral gyrus activity is reported more commonly. The M1 segment of the ipsilateral brain region and ventral premotor cortex are involved, although further studies are required to have a definitive conclusion [32].

A case study written by Ahdab et al. in 2020 [33] assessed the somatotopy within the primary motor cortex (M1). In this case, a 29-year-old man with recurrent strokes affecting finger muscles was studied. Motor mapping after the first cerebral attack revealed overlapping representations within M1 without evidence of distinct somatotopic organization. The hand knob region was spared in this incident. However, a subsequent stroke localized to the hand knob region resulted in significant selective weakness to the first dorsal interosseus muscle while sparing the abductor digiti minimi. The significance of this finding contradicts the initial mapping. Also, it emphasizes the nuanced organization of M1, where each muscle correlates to a unique area with limited ability for alternate pathways to compensate. Moreover, it is understood that the medial fingers and proximal joints are connected to the medial aspect of the hand knob region, whereas the lateral fingers are represented laterally. Understanding these organizations is crucial in addressing motor deficits and formulating the best treatment plan for patients [33].

It is noteworthy that, in this review, we mainly found case reports and articles that focused on the localizationist theory for explaining the mechanisms of knob st stroke [34]. In this context, neurological deficits are explained by damage to functionally specialized brain regions. However, experimental studies in primates about brain plasticity after stroke have shown that the brain is a densely interconnected network [35]. For example, the corticomotoneuronal system has been characterized as monosynaptic connections from cortical neurons in the primary motor cortex (M1; Brodmann’s area 4) to spinal motoneurons and is essential for the generation of finger movements and the fractionation of hand movements. However, the corticomotoneuronal output from M1 is not limited to hand muscles but also includes connections with the motoneurons of more proximal limb muscles. In this way, local necrosis of the brain tissue will also impact connected areas distant from the stroke lesion. Especially in hand knob stroke, further studies using functional MRI are needed to study deficits as well as recovery of the patients, focusing on the perspective of network-wide processes [36].

#### 3.3.2. Changes in Motor Function after Stroke

Hamzei et al. studied cortical reorganization after constraint-induced movement therapy, a type of motor rehabilitation, in stroke patients using functional MRI and transcranial magnetic stimulation. Patients with intact M1 regions and descending motor fibers had decreased activation of the sensory-motor cortex ipsilateral to the lesion, but intracortical excitability was higher. The converse was also true, indicating reorganization involving other brain regions [34]. A “recruitment activation pattern” of the bilateral sensory-motor cortices, premotor cortex, and supplementary motor area has been observed in patients with M1 lesions in previous studies [35].

Motor rehabilitation is key after a stroke to improve the chances of functional recovery. Functional imaging techniques have been used to demonstrate that changes in cortical activity occur with clinical motor improvement. A study by Jang et al. found a correlation between motor recovery with increased sensory-motor cortex activity of the ipsilateral hemisphere along with increased size in individuals with hand knob infarct [37].

Jang et al., 2005 demonstrated an improvement in hand motor function associated with sensory cortex involvement using functional MRI. The hand knob area was infarcted in both subjects studied, and recovery was over several months, which was attributed to neuroplasticity. The reorganization of the cortex surrounding an infarcted region is a valid explanation for plasticity. An intact cortical spinal tract originating in the premotor cortex, the supplementary motor area, and the parietal lobe is required for the above-described reorganization [38].

Reorganization of both motor and sensory function into a perilesional area following a stroke has been described for the first time by Jang et al. A patient with a right-sided hand knob area infarct recovered function 6 months later. Also, the functional MRI demonstrated activation of the lateral area of the infarct when a special apparatus was used to elicit active and passive movements. Previous studies have also demonstrated the reorganization of motor function into posterior and lateral regions of the infarcted area. Literature reports that cortical strokes had better functional recovery than strokes affecting the corona radiata and posterior limb of the internal capsule, suggesting a lack of perilesional reorganization in these regions [39].

Connectivity of specific regions of the brain, such as the ipsilateral (to the lesion) basal ganglia, thalamus, medullary pyramid, and contralateral cerebellum, have been linked to the improvement of motor function in patients with intracerebral hemorrhage resulting in hemiparesis [40]. Diffusion tensor imaging using probabilistic tracking was utilized to establish connections between these brain regions and improve hand motor function. The corticospinal tract and the extramedullary pyramids play key roles in motor function [41].

A meta-analysis performed by Rehme et al. provided a comprehensive understanding of neuronal activity post-stroke. Prior studies had limitations, including limited power, low reliability of imaging techniques, and potential confounding factors blurring interpretation. The authors described increased activation of bilateral premotor cortices and contralateral (to the lesion) motor area post-stroke. This activity depends on several factors, including the degree of motor impairment and the time after stroke [42].

In 2016, Jang et al. investigated injury to the corticofugal tract through diffusion tensor tractography (DTT) in limb-kinetic apraxia among subjects with a history of corona radiata infarct. Using DTT, researchers could reconstruct the corticospinal tract and the corticofugal tract. These tracts are neural pathways responsible for voluntary motor control. Fractional anisotropy, mean diffusivity, and tract volume were measured for both tracts. The authors found that there were significant decreases in fractional anisotropy and tract volumes in the hemisphere affected by the infarct when compared to the unaffected hemisphere. Also, the findings were notable within the precentral hand knob distribution of the corticospinal tract, with the study concluding that limb-kinetic apraxia as a result of damage to the corticofugal tract may also be joined by damage to the corticospinal tract as well [43].

## 4. Clinical Assessment, Management, and Prognosis

### 4.1. Clinical Assessment

Stroke results in spasticity in as high as 50 percent of the patients, and spasticity worsens over time or remains stable in most cases. Severe spasticity is a contributing factor negatively affecting the sensorimotor prognosis along with weakness and lesion size on the corticospinal tract. Spasticity is also associated with a higher occurrence of contractures and pain, highlighting the need for improved care and the potential use of botulinum toxin [43]. Plantin et al. assessed flexion resistance in the wrist and fingers after stroke. The authors noticed a correlation between the flexion resistance soon after the stroke and long-term complications. Interestingly, they also found that hand spasticity measurements were associated with the prediction of overall motor recovery [44].

Transcranial magnetic stimulation using neuronavigational and neuroanatomical images can provide motor maps that are helpful for the development of specific techniques for management. Giuffre et al. assessed healthy individuals for the anatomical “hand knob”, and they observed that motor mapping is relatively reliable and that smaller map percentile subsets showed decreased variability but only minimal improvements in reliability. However, the minimal detectable change for most transcranial magnetic stimulation mapping outcomes was above 50 percent, so neuroplasticity over time should be considered, and corrections in the mapping over time should be done [45]. Some authors even proposed that the wide variety of effectiveness of transcranial magnetic stimulation in clinical trials can be associated with a specific determined neuroanatomical area, and they propose that every individual should have an individualized configuration of the stimulation settings [46]. The main concerns regarding individualized maps are that no specific evaluation of the rehabilitation techniques can be assumed, and the sample size of the studies should be increased to overcome the confounding variables.

Maeder-Ingvar et al. studied only strokes presenting with monoparesis. They observed that most of them have an ischemic origin and a favorable prognosis. Also, the authors found that the neurological deficit occurred due to lesions localized in the superficial middle cerebral artery. Moreover, patients with monoparesis, when compared to hemiparesis, have a lower number of risk factors for stroke [47]. Better outcomes in monoparesis may be explained by fewer risk factors, suggesting individuals with decreased neuroinflammation and lower limitations regarding other comorbidities. Also, another hypothesis could be the individualized rehabilitation techniques for specific structures when compared to body segments.

### 4.2. Management Subsection

Lambercy et al. developed knobs for hand rehabilitation of patients with stroke based on their specific features, and the authors noticed increased augmented somatosensory information, including proprioceptive and tactile stimuli. Lambercy et al. proposed that spasticity could be associated with abnormal patterns of muscle activation following stroke, and this may be reduced with knobs involving proprioception [48]. Some case reports describe the effectiveness of early task-specific and action observation mirror techniques in individuals with stroke of the hand knob area presenting with isolated wrist drops. It is believed that these techniques prevent compensatory movements, leading to further progression [49].

A significant proportion of stroke recovery is rehabilitation. The randomized clinical trial by Ang et al. demonstrated that using advanced techniques such as motor imagery-brain-computer interface has shown promising results in objectively improving proximal and distal muscle functional recovery in stroke patients. Also, the study proposed a brain-computer interface coupled with a haptic knob robot combined with therapist-assisted arm rehabilitation. However, further studies with larger sample sizes are needed to improve generalizability [50].

Continuous theta burst stimulation using transcranial magnetic technique can perturbate the putative cortical area, contributing to functional recovery. Roux et al. found that this specific stimulation is limited by the variability of human motor maps and the cortical vicariation mechanisms, in which one cortical area is replaced by another [51]. To overcome this barrier, Kim et al. studied the difference in the outcomes of individuals according to stimulation of the hand knob versus induced hand motor hotspots [52]. The motor hotspot was determined by the area related to the major concentration of motor-evoked potentials in the contralateral first dorsal interosseous muscles. They noticed that the induced hotspot, when compared to the anatomical hand knob area, was more effective and was an optimal target area to induce low frequency [53].

Some authors proposed the combination of repetitive facilitative exercise and low-amplitude continuous neuromuscular electrical stimulation to recover post-stroke pure motor-isolated hand palsy. Both techniques can be used to promote neurofacilitation and neuroplasticity at the same time, which promotes rehabilitation in a short period of time [54]. Also, favorable outcomes can be associated with the high number of individual finger active-movement repetitions. Interestingly, some scales performed were the Fugl-Meyer assessment for the upper extremity, the action research arm test, the simple test for evaluating hand function, and the motor activity log.

Another possible approach for patients with impairment of wrist flexion and stroke is an epidural implant capturing the electrocorticographic brain activity. Beta burst stimulation onto the hand knob anatomical region was performed, and the epidural field potentials were recorded. This technique proved more useful than recording field potentials of attempts to move a paralyzed hand [55]. Also, the epidural implants have a high spatial resolution when compared to electroencephalographic approaches. However, epidural implants, when compared to surface electroencephalography, may be associated with a higher number of side effects because their disposition involves a significant number of structures.

Modified Brinkman board and reach and grasp drawer tasks were performed in macaque monkeys who underwent dorsolateral prefrontal cortex biopsy and autologous adult neural cell ecosystem. Badoud et al. observed no significant changes in the brain biopsy after appropriate training [56]. Interestingly, similar findings were also noticed in functional MRI performed to predict human grip force [57].

### 4.3. Prognosis

Turton et al. investigated the hand function in individuals with stroke, and they observed that task-specific training does not improve latencies or the clinical function of the hand in electrodiagnostic studies [58]. On the other hand, List et al. found that cortical thickness and coil-to-cortex distance of the hand knob were indirectly related to cortical excitability and learning ability. Thus, these two parameters can be used to predict functional recovery after stroke of the hand knob region [59].

In studies involving perinatal stroke and remodeling of the motor cortex, it was observed that the motor network may be displaced to both hemispheres. Also, the displacement was even stronger in individuals with arterial ischemic strokes with significant ipsilateral control of the affected limb [60]. A possible neuroimaging feature correlated with hand function recovery in individuals with hypertensive intracerebral hemorrhage is diffusion tensor imaging. The Brunnstrom recovery stage of the hand was negatively correlated with the degrees of integrity of the corticospinal cord [61].

## 5. Peripheral Versus Central Wrist Drop

Stroke presentations of isolated motor symptoms are easily confounded with peripheral neuropathy [21]. In this context, discrete stroke in the parietal lobe or the white matter of the angular gyrus, ventral posterior thalamus, and the posterior limb of the internal capsule can mimic peripheral nerve lesions. Notably, individuals with cortical hand strokes are likely to have a high percentage of recurrence [3]. A careful history-taking and neurological examination should be obtained to decrease the significance of confounding factors.

**Figure 3:**
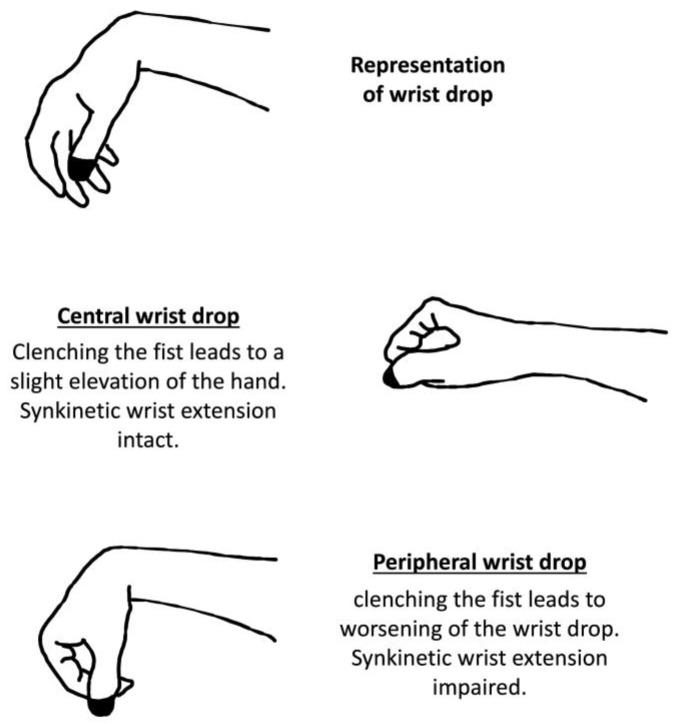
Synkinetic wrist extension in wrist drop.

Brigo et al. proposed the analysis of synkinetic wrist extension to differentiate cortical hand from peripheral nerve palsy (Figure 3). They observed that central wrist drops preserve synkinetic wrist extension of the long forearm extensors when clenching the fist, characterized by a slight hand elevation. A normal fist closure is associated with concurrent contraction of the long forearm, leading to the wrist in a neutral position with strong finger flexion [62]. On the other hand, in peripheral lesions, clenching the fist leads to worsening of the wrist drop.

Another neurological sign differentiating central from peripheral wrist drops is sensory. In central lesions, no dermatomal distribution is observed in sensory deficits, and in most patients, only motor symptoms without sensory symptoms are reported. However, some patients can report mild paresthesias that improve within days of the stroke.

### Other Unusual Presentations

Sensorimotor cortex evaluation with somatosensory evoked potential revealed a distinct somatotopic distribution of the human hand, from medial superior to lateral inferior, in the following order: ulnar nerve, median nerve, and then lip. There are some reports in the literature of ulnar palsy after a stroke of the hand knob area, which is known as pseudo-ulnar palsy [63]. It is worth mentioning that they usually have associated sensory deficits due to lesions in the sensory cortex and thalamus. Interestingly, patients can present with cortical symptoms like transient expressive aphasia [64]. Kakinuma et al. reported an interesting case of sensory and motor disturbances in the ulnar side of the right hand, in which the weakness progressed to the entire arm over three days [65].

Leet et al. reported a patient presenting with a right-hand wrist drop, which two years later developed into an isolated left-hand wrist drop. Patent foramen ovale etiology for his stroke was assumed after other etiologies were ruled out [66]. Several cases with selective palsy of a particular group of fingers without sensory deficits due to cortical infarction of the precentral knob have been reported. Still, isolated index finger palsy is rare [67]. Kim et al. investigated only this subgroup of individuals, and they observed that embolic mechanisms were more often associated with palsies of radial-side fingers than of ulnar-side fingers [13].

## 6. Limitations and Future Studies

There are some limitations in the present study. Most importantly, this is not a systematic review. The low incidence of hand knob stroke and the limited study of this condition prompted us to include case reports and case series in order to review the literature thoroughly. However, the incidence of this condition could be even higher than it is documented because the isolated presentation of hand palsy could be mistakenly diagnosed as peripheral neuropathy, for example. In this way, the clinical presentation and epidemiology of this condition could differ from the characteristics we found in this review. Future studies may focus on the long-term outcomes of patients with strokes in the hand knob region. Longitudinal studies on a larger scale with access to a wider and more diverse data set will be the next area of focus. Analyzing patient outcomes following rehabilitation programs can help us develop the most optimal and standardized treatment pathway. Continued investigation of imaging modalities such as MRI or diffusion tensor imaging can provide insight into the structural remodeling associated with hand knob stroke as well as map functional connectivity to other brain regions.

Neuroplasticity is an evolving idea, and studies in this area can be meaningful in rehabilitation efforts, not only for a hand knob stroke but for stroke in general. Identification of patient factors that impede presentation in the setting of isolated hand paralysis can help create awareness to mitigate future risks. Current rehabilitation research is promising, and efforts in this direction can be supported by longitudinal data provided by institutions serving patients with strokes. Researching the hand knob area and developing a compositional code with the use of a brain–computer interface appears to be a promising endeavor that needs further studies to reach a level of clinical utility. Future studies about the use of hand knob areas on imaging to teach students and residents can be helpful and understanding the impact of this anatomical landmark on medical education.

The hand motor cortex is remarkable for its anatomical reliability. In this way, rehabilitation models regarding stroke should be primarily tested in individuals affected by this specific location. The literature regarding different management techniques is vast, but no specific procedure has shown improvement in stroke outcomes in a large number of patients. In this way, the hand motor cortex should be individualized, and hotspots should be rendered before the start of the rehabilitation.

The literature about the prognosis of stroke of the hand knob area is scarce. This is a common question for patients and their family members in the acute phase of stroke. Future studies should analyze the main factors associated with the recovery outcomes and construct medical calculators for the development of the current knowledge.

## 7. Conclusions

Strokes affecting an omega-shaped region of the precentral gyrus, an anatomical landmark area known as the hand knob, are often confused with peripheral nerve palsies. The hand knob area holds significance in rapidly growing subdomains of neurology and neurosurgery, including functional neuroscience and rehabilitation. Strokes affecting the hand knob area constitute less than one percent of strokes, and it is essential to have an algorithmic approach while dealing with a presentation. The acute presentations lead to prompt diagnosis, but the subacute forms may lead to delays in the diagnosis and high costs related to the diagnostic tools. The most common etiologies for a stroke in this area can be attributed to large vessel occlusion, small vessel occlusion, and cardioembolism. Understanding of hand knob strokes can lead to further improvement in the development of techniques related to rehabilitation. Further studies are required to evaluate the prevalence of hand knob strokes, and reporting further cases can be helpful to the scientific community.

## Figures and Tables

**Table 1:** Literature review of reports of hand knob stroke in Pubmed/Medline.

Reference	Demographic Data (Sex/Age)	Ischemic Stroke Type (TOAST Criteria)	Outcome	Note
Tei et al. (1999) [1]	75/M	Likely small vessel occlusion of hand knob area	Weakness of distal right arm, especially with fine movements. Stereognosis, position sense, and sensation were intact	Cranial computed tomography did not reveal findings of the acute stroke
Back et al. (2001) [2]	81/F	Likely small vessel occlusion of cortical precentral hand knob area	Flaccid paralysis of the right arm without loss of sensation or reflexes	-
Gass et al. (2001) [3]	73/M	70% stenosis of right ICA, LVO	DAP + facial and wrist palsy, mild sensory loss	Weakness was measured according to the medical research council scale
	44/F	Dissection of left ICA, other determined etiology	DAP + ulnar involvement	
	62/F	Undetermined etiology- likely cardioembolic as workup revealed cardiac arrhythmia	DAP + facial palsy and mild sensory loss	
	74/M	Right RCA stenosis 80%, LVO	DAP	
	43/M	Large PFO; other determined etiology	DAP + radial nerve symptoms	
	59/M	Left ICA stenosis 80%, LVO	DAP + facial, wrist, elbow palsy with mild sensory loss	
	79/F	80% stenosis of right ICA, LVO	DAP + ulnar nerve palsy symptoms	
	75/M	No occlusion, undetermined etiology	DAP + ulnar nerve palsy	
	49/F	L MCA stenosis, LVO	DAP	
	69/F	Undetermined etiology	DAP	
	76/M	Likely cardioembolic as the patient had PFO	DAP + radial nerve palsy symptoms	
	75/F	Likely cardioembolic, as the patient had cardiac arrhythmia	DAP + radial symptoms	
	66/M	Other undetermined etiology	DAP + ulnar nerve symptoms	
	77/M	Likely cardioembolic in the setting of PFO	DAP + wrist and elbow palsy	
Granziera et al. (2008) [4]	67/M	Cardioembolism	Isolated left-hand paresis with paresis of thumb and index flexion	-
Hall et al. (2008) [5]	67/M	Right internal carotid plaque (large artery atherosclerosis)	Plegia of left-hand flexion and extension	-
Peters et al. (2009) [6]	70.8 (mean)/29 subjects (22M and 7F)	Atherosclerosis of the carotid artery (27/29 subjects), large artery atherosclerosis. Cardioembolic source (4/29 subjects)	All 29 subjects with contralateral arm paresis were detected on diffusion-weighted imaging. 16 patients had right-sided infarcts, and 13 had left-sided infarcts	79% of the individuals had full recovery within 25 months
Kitamura et al. (2010) [7]	73/M	Cardioembolism	Pure motor isolated finger palsy of the left thumb and index finger	Some peripheral nerve palsies could be isolated motor strokes
Alstadhaug et al. (2013) [8]	62.61 (mean)/13 subjects (11M and 2F)	LVO noted in 11/13 subjects Two subjects had cardioembolism	Acute isolated hand paresis of contralateral arm	-
Dekeyzer et al. (2014) [9]	69/M	Stenosis of cervical and cavernous segment of R ICA, large vessel atherosclerosis	Paresis of the left arm, mild hyperreflexia, and lack of sensory symptoms	Non-contrast cranial CT scan demonstrated blurring of right-hand knob area
Jusufovic et al. (2015) [10]	44/F	Right proximal MCA stenosis, large vessel atherosclerosis	Motor deficits of left arm—claw hand deformity, impairment of adduction and abduction, brisk deep tendon reflexes	-
De Medeiros et al. (2017) [11]	71/F	Cardioembolic source as the patient had atrial fibrillation	Right-hand weakness, fine motor impairment, preserved reflexes, lack of sensory involvement	-
Dijkstra et al. (2017) [12]	78/M	-	Weakness of right thumb and index finger	-
Kim et al. (2017) [13]	60 (mean)/17 subjects (8M and 9F)	-	-	-
Folyovich et al. (2018) [14]	70/M	Pulmonary adenocarcinoma metastasis to precentral gyrus, stroke of other determined etiology	Left upper extremity weakness without sensory loss	Left arm weakness was the presenting sign of the malignancy
Orosz et al. (2018) [15]	67 (mean)/25 subjects (12M and 13F)	12/25 were secondary to large artery atherosclerosis. Nine were strokes of undetermined etiology, four were strokes of determined etiology including anus carcinoma, carotid artery dissection, lupus anticoagulant, and factor V Leiden	Arm paresis contralateral to the area affected	One subject had bilateral hand paresis secondary to bilateral hand knob infarctions
Wang et al. (2018) [16]	67 (mean)/9 subjects (4F and 5M)	Three subjects developed large artery atherosclerosis. Three subjects developed stroke of undetermined etiology. Two subjects developed cardioembolism. One subject developed stroke of other determined etiology (Moya Moya affecting bilateral MCA)	Distal arm weakness, one subject with radial features, one subject with ulnar features and the rest of them with uniform features	Lesions of the lateral hand knob area affected radial nerve distribution, and lesions of the medial hand knob area affected ulnar nerve distribution indicating a topographic representation. This study is the first report of Moya Moya disease causing hand knob infarction
Finkelsteyn et al. (2019) [17]	60 (mean)/12 subjects (9M and 3F)	Two subjects developed large artery atherosclerosis (ipsilateral ICA stenosis greater than 50%). Two subjects developed cardioembolism (one with intracardiac thrombus and one with AV block). One subject developed a stroke of other determined etiology (thrombophilia). Seven developed strokes of undetermined etiology	Contralateral arm paralysis without sensory findings	Largest cohort of patients with hand knob area stroke in Latin America
Tomoda et al. (2019) [18]	72/M	Thromboembolism secondary to pancreatic adenocarcinoma resulting in hypercoagulable state, stroke of other determined etiology	Paresis of left arm extension	Cancer-associated embolism has a high rate of recurrence
Shelley et al. (2020) [19]	59/M	Hyperhomocysteinemia, stroke of other determined etiology	Inability to extend right wrist and finger (isolated right pseudo radial paralysis)	-
	47/M	Hyperhomocystinemia resulting in common carotid artery thrombus, stroke of other determined etiology	Left pseudo-radial paralysis	-
	30/F	Metastatic lesion to the hand knob area, from primary adenocarcinoma of the lung, stroke of other determined etiology	Left pseudo-median paralysis	-
	60/F	Giant cell arteritis, stroke of other determined etiology	Left pseudo-median paralysis	-
	35/M	Common carotid artery thrombosis in the setting of iron deficiency anemia, thrombocytosis, stroke of other determined etiology	Right pseudo-radial paralysis	-
	65/F	Left ventricle thrombus that embolized to left ICA (artery to artery embolism), stroke of other determined etiology	Right pseudo-median palsy	First known case of artery-to-artery embolism resulting in hand knob infarct
Daneasa et al. (2021) [20]	106/M	Stenosis of the left internal carotid artery, large artery atherosclerosis	Distal paralysis of the right hand	The oldest patient reported in the literature with hand knob infarct
Davies et al. (2022) [21]	59/M	Unknown mechanism in both cases, possible large artery atherosclerosis	Sudden onset of left-hand grip strength loss	Highlights the importance of recognition of stroke symptoms by emergency department physicians and discusses the risk of future stroke
	88/M		Sudden painless weakness of left hand	
Nicolini et al. (2022) [22]	83/M	Cerebral air embolism in the setting of central venous catheterization that traveled through a PFO, stroke of other determined etiology	Sudden, transient loss of consciousness, left arm weakness upon waking up	First ever reported case of cerebral air embolism resulting in hand knob infarct
Zhang et al. (2022) [23]	62 (mean)/9 subjects (5M and 4F)	Six subjects with large artery atherosclerosis, six subjects with small artery occlusion, one subject with cardioembolism, two subjects with a stroke of undetermined etiology, and two with strokes of other determined etiology	Contralateral hand paresis in all 19 subjects	Largest cohort of patients with hand knob infarct in China
Alshanqiti et al. (2023) [24]	62/F	Fibromuscular dysplasia and severe atherosclerotic stenosis of the right internal carotid artery (large artery atherosclerosis versus stroke of other determined etiology)	Wrist extension drop of the left hand, no sensory deficits	First known report of hand knob infarct secondary to fibromuscular dysplasia

Abbreviations: DAP, distal arm paralysis; F, female; ICA, internal carotid artery; LVO, large vessel occlusion; M, male; MCA, middle cerebral artery; PFO, patent foramen ovale; TOAST, Trial of Org. 10172 in Acute Stroke Treatment.

**Table 2:** Epidemiological studies of hand knob stroke.

First Author et al./Year	Country	Incidence	Number of Individuals Affected	Number of Individuals in the Study
Finkelsteyn et al. (2019) [17]	Argentina	3.53%	12	339
Alstadhaug et al. (2013) [8]	Norway	1.5%	13	866
Celebisoy et al. (2007) [25]	Turkey	0.98%	8	815
Zhang et al. (2022) [23]	China	0.9%	19	2224
Peters et al. (2009) [6]	Germany	0.83%	29	3499
Orosz et al. (2018) [15]	Hungary	0.35%	25	720

## Data Availability

No new data were created or analyzed in this study. Data sharing is not applicable to this article.
